# Elevated atherogenic index and higher triglyceride increase risk of kidney function decline: a 7-year cohort study in Chinese adults

**DOI:** 10.1080/0886022X.2020.1853569

**Published:** 2020-12-13

**Authors:** Fei Huang, Le Wang, Qian Zhang, Zhengce Wan, Liu Hu, Ranran Xu, Anying Cheng, Yongman Lv, Qingquan Liu

**Affiliations:** a Department of Geriatrics, Tongji Hospital, Tongji Medical College, Huazhong University of Science and Technology, Wuhan, China; b Department of Nephrology, Tongji Hospital, Tongji Medical College, Huazhong University of Science and Technology, Wuhan, China; c Department of Health Management Centre, Tongji Hospital, Tongji Medical College, Huazhong University of Science and Technology, Wuhan, China

**Keywords:** Atherogenic index of plasma, triglyceride, glomerular filtration rate, risk factors

## Abstract

**Objective:**

This study explored whether lipid disorders or an elevated atherogenic index of plasma (AIP, a risk factor for cardiovascular diseases) could predict major kidney function decline.

**Methods:**

We conducted a retrospective 7-year cohort study of 3712 Chinese adults followed up between 2010 and 2017. Major kidney function decline was defined as *a* ≥ 30% reduction in the estimated glomerular filtration rate (eGFR) from baseline. Multivariable logistic regression models were used to evaluate the relationship between lipid profiles and major kidney function decline. Smoking habits, waist circumference, and physical activity were not assessed.

**Results:**

During the 7-year follow-up, 1.70% (*n* = 63) of the participants developed major kidney function decline. After adjustment for potential confounders, the odds ratios (ORs) for developing eGFR decline with per standard deviation increase were 1.23 [95% confidence interval (CI): 1.06–1.43] for triglyceride and 2.55 (95% CI: 1.01–6.42) for AIP in all participants. Furthermore, in the stratified analysis, we found sex-related differences; triglyceride and AIP were only independently associated with the risk of eGFR decline in men (OR, 1.27, 95% CI: 1.08–1.48; OR, 3.98, 95% CI: 1.22–12.99, respectively). When the participants were divided into groups according to the baseline lipid status, association was observed only between abnormal AIP and eGFR decline (all *p* values < 0.05).

**Conclusion:**

Our findings suggest that a higher serum triglyceride level or an elevated AIP increases the risk of major kidney function decline in Chinese men with normal kidney function. Thus, assessment of AIP may help identify the risk of eGFR decline.

## Introduction

Chronic kidney disease (CKD) is now a major public health concern worldwide and is associated with end-stage renal disease as well as cardiovascular morbidity and mortality [1–4]. The prevalence of CKD is estimated to be 8%–16% in the general adult population [5] and nearly 10.8% in China [2]. A decline in the estimated glomerular filtration rate (eGFR) might be associated with adverse outcomes and CKD development, even among those people with normal kidney function. Thus, management of modifiable eGFR decline risk factors is crucial for preventing disease incidence.

Lipid disorders (such as dyslipidaemia) have been identified as critical contributing factors for atherosclerosis and cardiovascular disease (CVD) [6]. Previous epidemiologic observations have suggested an independent association between serum lipids and CKD development [7–10] and usefulness of lipid-lowering therapy in slowing the rate of CKD progression [11–13]. Interestingly, conclusions regarding the association between serum lipids and CKD development are divergent and conflicting [14–17], which might be caused by the different ethnicities of the groups studied. Meanwhile, dyslipidaemia might have a greater influence on CKD development in men than in women [18,19]. Atherogenic index of plasma (AIP), calculated as the logarithmically transformed ratio of triglyceride (TG) to high-density lipoprotein cholesterol (HDL-C) (lg[TG/HDL-C]), is a novel marker of atherosclerosis and CVD. In addition, related studies have shown AIP to be a more accurate predictor of CVD than traditional lipid parameters [20–23]. However, only a few studies have investigated the association between AIP and eGFR decline or have revealed the potential clinical usefulness of AIP [24]. Most studies investigating the association of serum lipids with kidney function have examined only patients with impaired kidney function (eGFR < 60 mL/min/1.73 m^2^). A better understanding on the relationship between potential risk factors and eGFR decline in the general population with a completely normal kidney function is of great clinical interest.

Therefore, this study investigated the independent association of serum lipid profiles as well as AIP with the development of major kidney function decline and presented potential sex-related differences in a cohort study in China. The definition of major kidney function decline and follow-up period was set based on previous studies [25,26].

## Materials and methods

### Participants

**Figure 1.**
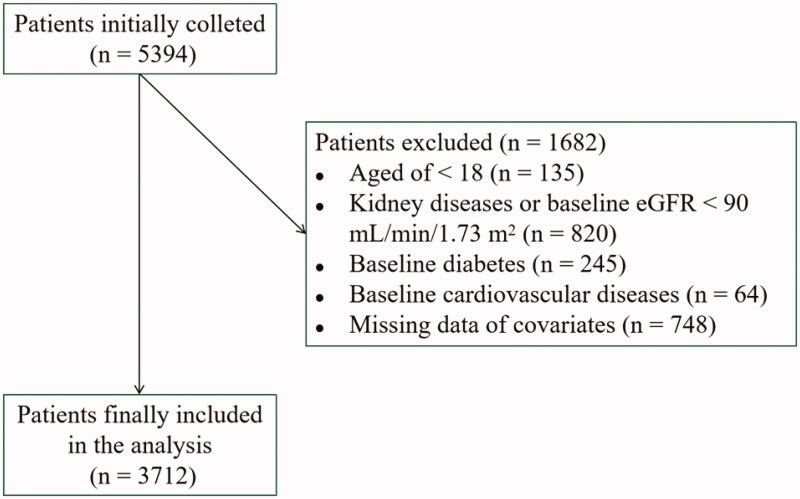
Flowchart of the study participants.

In the present study, we enrolled participants aged 18–85 years who had undergone a comprehensive medical examination at baseline (2010) and whose results were reevaluated 7 years later (2017), with both examinations conducted at the health manage center of Tongji Hospital (Wuhan, China). All participants were of Chinese ethinicity. Initially, 5393 individuals were identified as potential participants. The exclusion criteria were as follows: (1) baseline eGFR < 90 mL/min/1.73 m^2^ (*n* = 820); (2) past history of CVD (*n* = 64) or past history of diabetes (*n* = 245) at baseline; and (3) missing data for any of the study variables (*n* = 748). Finally, a total of 3712 eligible participants (2008 men and 1704 women) were included for our analysis. The study was approved by the Ethics Committee of Tongji Hospital, Tongji Medical College, Huazhong University of Science and Technology (The Institutional Review Board Approval Number: TJ-C20160115). The study conforms to the principles outlined in the Declaration of Helsinki, and written informed consent was obtained from all participants (Figure 1).

### Anthropometric and biochemical measurements

Routine anthropometric measurements were performed by trained examiners. Height and weight were measured to the nearest 0.1 cm and 0.1 kg, respectively, while the participants were asked to wear light clothing and stand barefoot. Body mass index (BMI) was calculated as weight (kg)/height (m)^2^. After the participants rested quietly for a minimum of 5 min, systolic and diastolic blood pressures (SBP and DBP, respectively) were measured using a standard electronic sphygmomanometer (HBP-9020; OMRON, Dalian, China). Data on sex, age, ethnicity, and individual history of disease were collected using a questionnaire. Fasting (over 12 h) blood samples were collected from each participant and sent to the clinical laboratory of Tongji Hospital for standard biochemical measurement within 2 h of collection. Total cholesterol (TC), TG, low-density lipoprotein cholesterol (LDL-C), HDL-C, fasting plasma glucose (FPG), uric acid, and creatinine were determined using standard laboratory procedures.

### Criteria and definitions

As a study focused on GFR decline, the outcome major kidney function decline was defined as *a* ≥ 30% reduction in the eGFR from baseline to a value of <90 mL/min/1.73 m^2^. This criterion was set based on the previous study conclusion that a 30% reduction in the eGFR could be recommended as an alternative end point for CKD progression [25]. The 7 year period was set based on Young’s study which used a similar length of follow-up to define new-onset CKD [26]. The eGFR was calculated using the CKD-EPI equation [27]. For men with Scr (in mg/dL) ≤ 0.9, eGFR = 141 × (Scr/0.9)^−0.411^ × 0.993^Age^, and for men with Scr > 0.9, eGFR = 141 × (Scr/0.9)^−1.209^ × 0.993^Age^. For women with Scr ≤ 0.7, eGFR = 144 × (Scr/0.7)^−0.329^ × 0.993^Age^, and for women with Scr > 0.7, eGFR = 144 × (Scr/0.7) ^−1.209^ × 0.993^Age^. In our study, the cutoff values for higher TC, higher TG, higher LDL-C, and lower HDL-C were 6.22, 2.26, 4.14, and 1.04 mmol/L, respectively [28]. AIP is believed to be a useful independent predictor of CVD [29]. In the current study, we also used AIP to predict the risk of major kidney function decline. AIP > 0.15 was regarded as abnormal [29]. We also divided the 2008 male participants and 1704 female participants into tertiles. Tertile 1 (T1) had the lowest mean AIP, and Tertile 3 (T3) had the highest AIP.

### Statistical analysis

Data are expressed as means ± standard deviation (SD) for normally distributed variables, and as median with IQR for non-normally distributed data. Categorical variables are expressed as frequencies. Comparisons between two groups were examined using the independent *t*-test, Mann–Whitney *U* test, or chi-square test where appropriate. Multivariable logistic regression models were used to determine the relationship between lipid profiles and the prevalence of major kidney function decline, with data presented as odds ratios (ORs) and 95% confidence intervals (CIs). In this study, we used two models to adjust potential confounders for major kidney function decline. Model 1 was adjusted for age and sex. Model 2 included the variables of Model 1 and baseline BMI, FPG, uric acid, eGFR, SBP, and DBP. All statistical analyses were performed using SPSS software version 17.0 (SPSS Inc, Chicago, IL, USA). Two-tailed *p* values of <0.05 were considered statistically significant.

## Results

### General characteristics of the participants

Table 1 presents the general characteristics of the participants. A total of 3712 subjects were included in the study, and 54.1% of the participants were male. The mean age of the study population was 40.55 years. After a 7-year follow-up, 63 participants (1.70%) developed major kidney function decline. The mean annual eGFR decline was 1.8 mL/min/1.73 m^2^. The male participants had a higher BMI, a lower level of HDL-C, higher levels of blood pressure, TG, TC, LDL-C, AIP, FPG, creatinine, and uric acid, and a higher prevalence of hypertension (all *p* values < 0.05). No significant difference was observed in age. The data of participants in 2017 was presented as supplemental table 1.

**Table 1.** Baseline characteristics of participants relative to development of major renal function decline during the 7-year follow-up period.

	Overall	Men	Women	*p* Value
n	3712	2008	1704	
Age (years)	40.55 ± 10.74	40.82 ± 9.95	40.23 ± 11.58	0.102
BMI (kg/m^2^)	23.60 ± 3.61	24.72 ± 3.07	22.28 ± 3.74	<0.001
SBP (mmHg)	121.07 ± 17.05	124.88 ± 16.64	116.59 ± 16.44	<0.001
DBP (mmHg)	77.31 ± 11.88	80.80 ± 11.60	73.20 ± 10.86	<0.001
TC (mmol/L)	4.62 ± 0.88	4.68 ± 0.87	4.55 ± 0.89	<0.001
TG (mmol/L)	1.05 (0.72–1.61)	1.31 (0.92–1.90)	0.81 (0.61–1.16)	<0.001
HDL-C (mmol/L)	1.34 ± 0.32	1.21 ± 0.26	1.50 ± 0.31	<0.001
LDL-C (mmol/L)	2.77 ± 0.75	2.89 ± 0.73	2.63 ± 0.74	<0.001
AIP	−0.07 ± 0.32	0.06 ± 0.29	−0.23 ± 0.27	<0.001
FPG (mmol/L)	5.10 ± 0.52	5.15 ± 0.53	5.05 ± 0.50	<0.001
UA (mg/dL)	310.88 ± 84.32	362.25 ± 70.54	250.34 ± 53.40	<0.001
SCr (μmol/L)	65.62 ± 12.68	74.88 ± 8.10	54.71 ± 7.28	<0.001
eGFR (mL/min/1.73 m^2^)	110.20 ± 10.79	107.81 ± 9.53	113.01 ± 11.48	<0.001
Hypertension, n (%)	785	563 (28.0)	222 (13.0)	<0.001

Data are mean ± SD, median (IQR) or percentage.

BMI: body mass index; SBP: systolic blood pressure; DBP: diastolic blood pressure; TC: total cholesterol; TG: triglyceride; LDL-C: low-density lipoprotein cholesterol; HDL-C: high-density lipoprotein cholesterol; AIP: atherogenic index of plasma; FBG: fasting plasma glucose; UA: uric acid; SCr: serum creatinine; eGFR: estimated glomerular filtration rate.

### Logistic regression analyses for associations of serum lipids and AIP with major kidney function decline risk

Univariate and multivariable logistic regression models were used to determine the relationship between lipid profiles and the major kidney function decline risk (Table 2). In unadjusted models, TG and AIP were significantly associated with major kidney function decline in all participants. After adjustment for age and sex (Model 1), each 1 − SD increase in TG and AIP was associated with 1.28-fold (95% CI: 1.11–1.46) and 2.93-fold (95% CI: 1.27–6.76) increases in the risk of major kidney function decline, respectively. In the fully adjusted model (Model 2), the associations between TG and AIP and the prevalence of major kidney function decline were not materially changed (OR, 1.23, 95% CI: 1.06–1.43; OR, 2.55, 95% CI: 1.01–6.42, respectively) (all *p* values < 0.05).

**Table 2.** Adjusted ORs and 95% CIs for the presence of major renal function decline according to baseline serum lipids levels and AIP.

	Unadjusted		Model 1		Model 2	
	OR (95% CI)	*p* Value	OR (95% CI)	*p* Value	OR (95% CI)	*p* Value
Total						
TG	1.26 (1.10–1.43)	0.001	1.28 (1.11–1.46)	<0.001	1.23 (1.06–1.43)	0.006
TC	1.03 (0.78–1.36)	0.833	1.03 (0.77–1.39)	0.844	1.02 (0.75–1.38)	0.907
HDL-C	0.79 (0.36–1.75)	0.559	0.78 (0.32–1.92)	0.591	0.85 (0.33–2.19)	0.738
LDL-C	0.79 (0.56–1.12)	0.186	0.76 (0.53–1.10)	0.151	0.78 (0.54–1.14)	0.196
AIP	2.39 (1.14–5.00)	0.021	2.93 (1.27–6.76)	0.012	2.55 (1.01–6.42)	0.047
Men						
TG	1.27 (1.10–1.47)	0.001	1.28 (1.10–1.48)	0.001	1.27 (1.08–1.48)	0.003
TC	1.02 (0.70–1.50)	0.922	1.05 (0.71–1.55)	0.804	1.08 (0.72–1.60)	0.714
HDL-C	0.93 (0.25–3.42)	0.914	0.94 (0.26–3.49)	0.930	0.94 (0.23–3.85)	0.933
LDL-C	0.70 (0.43–1.12)	0.137	0.71 (0.44–1.15)	0.161	0.76 (0.46–1.25)	0.283
AIP	3.47 (1.20–10.09)	0.022	3.73 (1.28–10.92)	0.016	3.98 (1.22–12.99)	0.022
Women						
TG	1.26 (0.84–1.91)	0.267	1.00 (0.98–1.04)	0.627	1.07 (0.64–1.81)	0.790
TC	1.04 (0.69–1.57)	0.858	0.95 (0.59–1.52)	0.825	1.91 (0.56–1.48)	0.694
HDL-C	0.67 (0.19–2.30)	0.526	0.69 (0.20–2.35)	0.554	0.80 (0.22–2.89)	0.737
LDL-C	0.89 (0.53–1.51)	0.674	0.79 (0.44–1.40)	0.416	0.75 (0.42–1.35)	0.339
AIP	2.04 (0.57–7.29)	0.271	1.83 (0.46–7.29)	0.393	1.29 (0.28–5.95)	0.746

Model 1: adjusted for baseline age and gender.

Model 2: adjusted for covariates in model 1 plus baseline BMI, FPG, uric acid, eGFR, SBP and DBP.

Each variable is expressed per SD and was analyzed in a separate regression model.

SD: standard deviation; CIs: confidence intervals; TC: total cholesterol; TG: triglyceride; LDL-C: low-density lipoprotein cholesterol; HDL-C: high-density lipoprotein cholesterol; AIP: atherogenic index of plasma; BMI: body mass index; FBG: fasting blood glucose; eGFR: estimated glomerular filtration rate.

To investigate whether the associations among serum lipids, AIP, and the occurrence of major kidney function decline vary by sex, we further conducted subgroup analyses. After multiple adjustments, the associations among elevated TG and AIP and increased risk of major kidney function decline were only observed in the male participants (OR, 1.27, 95% CI: 1.08–1.48, *p* = 0.003; OR, 3.98, 95% CI: 1.22–12.99, *p* = 0.022).

Furthermore, we observed similar results when the participants were divided into groups according to the baseline lipid status (Table 3). In the fully adjusted model, an elevated risk of major kidney function decline was observed in all participants with an abnormal AIP (OR, 2.18, 95% CI: 1.18–4.05, *p* = 0.013) and in the male participants with an abnormal AIP (OR, 2.16, 95% CI: 1.02–4.55, *p* = 0.044). However, no association was observed between higher TG and increased risk of major kidney function decline regardless of sex. In addition, in the female participants, dyslipidaemia was not associated with the prevalence of major kidney function decline.

**Table 3.** Adjusted ORs and 95% CIs for the presence of major renal function decline according to baseline dyslipidaemia and abnormal AIP.

	Unadjusted		Model 1		Model 2	
	OR (95% CI)	*p* Value	OR (95% CI)	*p* Value	OR (95% CI)	*p* Value
Total						
Elevated TG	2.17 (1.17–4.04)	0.014	2.26 (1.18–4.34)	0.014	1.74 (0.86–3.53)	0.123
Elevated TC	2.23 (0.95–5.23)	0.067	2.26 (0.95–5.39)	0.066	1.98 (0.81–4.83)	0.132
Reduced HDL-C	1.61 (0.91–2.85)	0.105	1.65 (0.90–3.04)	0.107	1.55 (0.82–2.94)	0.177
Elevated LDL-C	1.04 (0.32–3.34)	0.953	1.03 (0.32–3.34)	0.963	0.95 (0.29–3.14)	0.933
Abnormal AIP	2.17 (1.30–3.62)	0.003	2.43 (1.38–4.29)	0.002	2.18 (1.18–4.05)	0.013
Men						
Elevated TG	2.04 (0.97–4.28)	0.061	2.16 (1.02–4.59)	0.045	1.78 (0.77–4.11)	0.173
Elevated TC	2.70 (0.93–7.81)	0.067	2.81 (0.97–8.18)	0.058	2.49 (0.82–7.61)	0.109
Reduced HDL-C	1.35 (0.67–2.74)	0.400	1.35 (0.67–2.74)	0.400	1.34 (0.63–2.87)	0.451
Elevated LDL-C	1.05 (0.25–4.42)	0.951	1.07 (0.25–4.52)	0.927	1.07 (0.25–4.69)	0.925
Abnormal AIP	2.09 (1.07–4.11)	0.030	2.15 (1.10–4.22)	0.026	2.16 (1.02–4.55)	0.044
Women						
Elevated TG	2.93 (0.86–9.94)	0.085	2.70 (0.77–9.52)	0.121	2.02 (0.52–7.88)	0.310
Elevated TC	1.64 (0.38–7.05)	0.505	1.44 (0.32–6.49)	0.635	1.31 (0.29–6.00)	0.726
Reduced HDL-C	2.97 (1.00–8.75)	0.048	2.87 (0.97–8.49)	0.057	2.57 (0.83–7.96)	0.101
Elevated LDL-C	0.99 (0.13–7.46)	0.998	0.86 (0.11–6.62)	0.881	0.75 (0.10–5.92)	0.787
Abnormal AIP	3.09 (1.23–7.75)	0.016	2.98 (1.13–7.85)	0.028	2.41 (0.82–7.04)	0.107

Model 1: adjusted for baseline age and gender.

Model 2: adjusted for covariates in model 1 plus baseline BMI, FPG, uric acid, eGFR, SBP and DBP.

CIs: confidence intervals; TC: total cholesterol; TG: triglyceride; LDL-C: low-density lipoprotein cholesterol; HDL-C: high-density lipoprotein cholesterol; AIP: atherogenic index of plasma; BMI: body mass index; FBG: fasting blood glucose; eGFR: estimated glomerular filtration rate.

### Prevalence of major kidney function decline according to AIP tertiles at baseline

**Figure 2.**
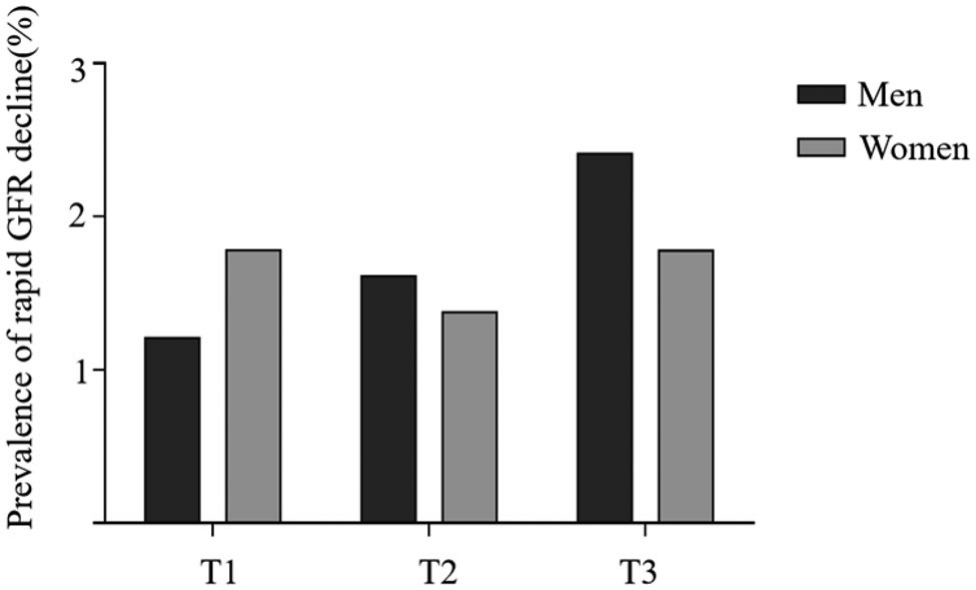
Adjusted prevalence of major kidney function decline according to the tertiles (T) of Atherogenic index of plasma (AIP).

After adjustment for age, the prevalence of major kidney function decline according to AIP tertiles was 1.2%, 1.6%, and 2.4% in the male participants and 1.7%, 1.3%, and 1.7% in the female participants (Figure 2). While the prevalence approximately doubled in the highest tertile than in the lowest tertile in the male participants, no significant linear trend was observed through the increasing tertiles in both sexes (*p* for trend = 0.09 in men, *p* for trend = in women). In addition, to verify the prediction efficiency of AIP, ROC curves analysis was generated for men with different outcomes (supplemental figure 1).

## Discussion

In this study focused on eGFR decline, after adjustment for potential confounders, higher serum TG and AIP were significantly associated with the development of major kidney function decline in men. In women, the serum lipids or AIP was not found to be a risk factor for major kidney function decline.

Mounting evidence has indicated that people with increased TG have a higher risk of CKD after adjustment for other risk factors [8–10,15]. In the present study, our results showed that baseline serum TG levels, but not other lipid parameters, had a strong positive association with major kidney function decline. In fact, the findings on the relationship between serum lipids and CKD development are inconclusive. Our results are similar to the conclusions of previous studies that TG levels are related to kidney function, which were also confirmed in the elderly population in Cao’s research [30]. Interestingly, some reports have presented a contradictory conclusion that no association exists between TG and kidney function [17]. Even in studies conducted in the Chinese population, the results are inconsistent [31,32]. The contrasting findings of these studies could be explained by the different characteristics of the study participants or differences in the study design. There is not much evidence to show the association between serum lipids and the development of major kidney function decline. Additionally, most previous studies on the association between serum lipids and kidney function have focused on patients with impaired kidney function (eGFR < 60 mL/min/1.73 m^2^) [14–16]. However, even a mildly decreased eGFR (60–74 mL/min/1.73 m^2^) has been reported to be associated with higher risk of CKD [33]. Our study showed that the associations between serum lipids and kidney function are also present in the participants with eGFR ≥ 90 mL/min/1.73 m^2^. In summary, the novelty of our study lies in the endpoint setting (major kidney function decline) and in extending the generality of the relationship to the general population with a completely normal kidney function.

In the stratified analysis, the association between higher TG levels and development of major kidney function decline was only observed in men. This sex-related difference was in line with the results of a previous study [18]. Zhang et al. found that serum TG was the only suitable predictor of CKD in men. However, in women, none of the serum lipids and the lipid ratio can be used as a predictor of CKD [18]. In fact, men have been reported to exhibit worse major kidney function decline progression than women [34,35]. However, the mechanisms underlying the sex-related difference in this association have not yet been defined. The difference in sex hormones and the sensitivity of their hormone receptors may affect the development of renal dysfunction through different pathophysiological pathways [34]. Female hormones might be involved in protecting women from CKD development.

Though smoking habits were not assessed in our study, it should be emphasized that smoking is closely associated with dyslipidaemia, especially higher TG and lower HDL-C [36]. In addition, research has shown the ability of smoking to modulate the postprandial hypertriglyceridemia, which represents the non-fasting TG levels and predicts the incidence of CVD [37]. Interestingly, evidence showed that metabolic effects of smoking on serum lipids might be altered by gender [38]. Therefore, the smoking data need to be gathered and quantified carefully in further studies.

AIP was first described by Dobiásová et al. [29] as a biomarker of plasma atherosclerosis. In their later study, AIP was found to be inversely correlated with the diameter of LDL-C particles and to be a surrogate for the small-dense LDL level [39], which is one of the major causative factors of arteriosclerosis and CVD [40]. In fact, a growing number of studies have suggested that AIP is a strong marker for predicting CVD risk [20,22,23,41]. A recent study showed that AIP is a predictor of subclinical renal damage, defined as an eGFR between 30 and 60 mL/min/1.73 m^2^ [42]. In our study, we found that high AIP was associated with an increased risk of major kidney function decline even after adjustment for multiple covariates. Our study further demonstrated that the association between AIP and renal dysfunction might exist in the general population with eGFR ≥ 90 mL/min/1.73 m^2^, and showed that AIP can not only predict CVD morbidity but also predict the development of renal dysfunction, which added to evidence indicating that CVD and CKD may have a similar pathophysiology. In fact, both the processes share certain pathophysiological mechanisms, such as endothelial dysfunction, increased oxidative stress, vascular ossification, and inflammation [43–45].

However, the mechanisms through which dyslipidaemia can potentially accelerate renal disease progression remain unclear. One of the possible mechanisms is that an increase in reabsorption of phospholipids and cholesterol by tubular epithelial cells is associated with dyslipidaemia. This reabsorption could then stimulate tubulointerstitial inflammation, foam cell formation, and tissue injury [46,47]. Moreover, increased levels of lipoproteins could increase the formation of proinflammatory cytokines [48], thus inducing glomerulosclerosis [49]. In addition, impaired renal function may accelerate lipid permeability and excretion in the glomerulus, further exacerbating dyslipidaemia in a vicious cycle [15].

### Limitations and strengths

Our study also has several limitations that merit consideration. First, this was a retrospective single-center study, which was unable to show whether the serum lipid profile is an independent risk factor for eGFR decline. Second, although albuminuria was also a crucial marker for kidney damage, we were unable to perform the analysis on albuminuria, as we did not have urinary albumin data at baseline and during the period thereafter. Evidence showed that the central obesity predicted the development of kidney injury [30], but waist circumference was not detected in this study. Other confounding factors, such as smoking and drinking habits and physical activity, were not assessed. Lastly, one important member in the lipid profiles, apolipoprotein, were not included. Given the previous evidence that increased apolipoprotein B might be associated with progression of CKD in diabetes patients [50], apolipoprotein should be considered in future studies.

Despite these limitations, the present study has several strengths. We excluded participants with known CVD and diabetes at baseline to eliminate the possibility of confounding due to concomitant diseases in relatively healthy individuals. In addition, we adjusted for many potential covariates, including age, BMI, FBG, uric acid, eGFR, SBP, DBP, which thus made our results more reliable.

## Conclusion

In summary, our findings showed that higher serum TG and AIP were significantly associated with the development of major kidney function decline in men. In women, none of the serum lipids or AIP was a risk factor for major kidney function decline. Further prospective studies are required to support our results and to compare the predictive values of individual lipid parameters with that of AIP in kidney function decline development.

## Supplementary Material

Supplemental Material

Supplemental Material
