# Supplementary figures and images for: Elevated atherogenic index and higher triglyceride increase risk of kidney function decline: a 7-year cohort study in Chinese adults
Source: Ren Fail. 2020 Dec 13;43(1):32–9. doi: 10.1080/0886022X.2020.1853569 (PMC7745844; doi:10.1080/0886022X.2020.1853569)

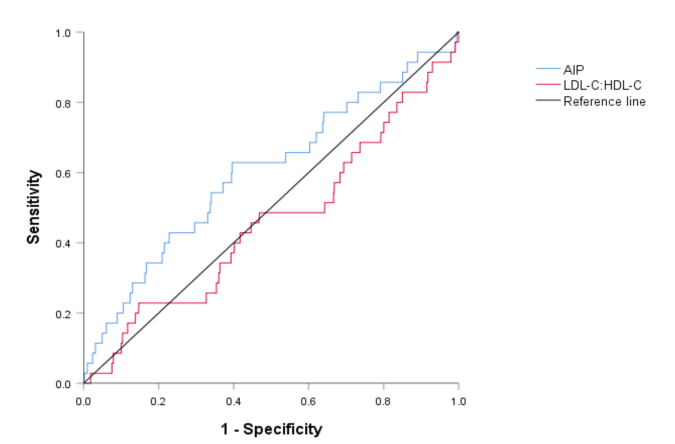

Supplement: Supplemental Material [file IRNF_A_1853569_SM4876.tif]
