# Supplementary material for: Elevated atherogenic index and higher triglyceride increase risk of kidney function decline: a 7-year cohort study in Chinese adults
Source: Ren Fail. 2020 Dec 13;43(1):32–9. doi: 10.1080/0886022X.2020.1853569 (PMC7745844; doi:10.1080/0886022X.2020.1853569)
Supplement: Supplemental Material [file IRNF_A_1853569_SM4848.pdf]

Supplemental table 1 Characteristics of participants in 2010 and 2017.

	2010 cohort	2017 cohort	*P* value
n	3712	3712	
BMI（kg/m^2）	23.60±3.61	23.94±3.21	<0.001
SBP（mmHg）	121.07±17.05	125.31±18.27	<0.001
DBP（mmHg）	77.31±11.88	77.70±12.29	<0.001
TC（mmol/L）	4.62±0.88	4.67±0.87	<0.001
TG（mmol/L）	1.05 (0.72-1.61)	1.25 (0.86-1.85)	<0.001
HDL-C（mmol/L）	1.34±0.32	1.30±0.31	<0.001
LDL-C（mmol/L）	2.77±0.75	2.79±0.74	0.164
AIP	-0.07±0.32	0.012±0.32	<0.001
FPG（mmol/L）	5.10±0.52	5.30±0.84	<0.001
UA（mg/dL）	310.88±84.32	340.59±92.58	<0.001
SCr（μmol/L）	65.62±12.68	72.70±14.08	<0.001
eGFR (mL/min/1.73 m^2）	110.20±10.79	98.11±12.88	<0.001
Hypertension, n（%）	785 (21.1)	1086 (29.3)	<0.001

Data are mean ± SD, median (IQR) or percentage.

BMI, body mass index; SBP, systolic blood pressure; DBP, diastolic blood pressure; TC, total cholesterol; TG, triglyceride; LDL-C, low-density lipoprotein cholesterol; HDL-C, high-density lipoprotein cholesterol; AIP, atherogenic index of plasma; FBG, fasting plasma glucose; UA, uric acid; SCr, serum creatinine; eGFR, estimated glomerular filtration rate.
